# DNA Strand Patterns on Aluminium Thin Films

**DOI:** 10.3390/s110706719

**Published:** 2011-06-28

**Authors:** Nadia Mahmoudi Khatir, Seyedeh Maryam Banihashemian, Vengadesh Periasamy, Wan Haliza Abd Majid, Saadah Abdul Rahman, Fatemeh Shahhosseini

**Affiliations:** 1 Low Dimensional Material Research Centre, Department of Physics, University of Malaya, 50603, Kuala Lumpur, Malaysia; E-Mails: vengadeshp@um.edu.my (V.P.); q3haliza@um.edu.my (W.H.A.M.); saadah@um.edu.my (S.A.R.); 2 Department of Physics, Islamic Azad University, Qom Branch, Qom, Iran; 3 Department of Genetics, Institute of Biological Sciences (ISB), University of Malaya, 50603, Kuala Lumpur, Malaysia; E-Mail: fatima2007@siswa.um.edu.my (F.S.)

**Keywords:** DNA pattern, nano-gap, DNA strands, Al thin film, etching

## Abstract

A new patterning method using Deoxyribose Nucleic Acid (DNA) strands capable of producing nanogaps of less than 100 nm is proposed and investigated in this work. DNA strands from *Bosenbergia rotunda* were used as the fundamental element in patterning DNA on thin films of aluminium (Al) metal without the need for any lithographic techniques. The DNA strands were applied in buffer solutions onto thin films of Al on silicon (Si) and the chemical interactions between the DNA strands and Al creates nanometer scale arbitrary patterning by direct transfer of the DNA strands onto the substrate. This simple and cost-effective method can be utilized in the fabrication of various components in electronic chips for microelectronics and Nano Electronic Mechanical System (NEMS) applications in general.

## 1. Introduction

Nanolithography based on X-ray exposure through a photomask on a photosensitive material and ion beam lithography are techniques used for nano- and microelectronic device fabrication [1,2]. Nanotransfer printing or stamping is another method for patterning on the surface of metals and semiconductors [3,4].

In 1994 Perkins [5] and Snow [6] independently investigated the AFM method of nanopatterning on substrates based on a chemo-mechanical mechanism. Later in 2005 Headrick *et al.* [7] reported that alkyl monolayer-coatings could be used to etch nanoscale patterns on silicon (Si) substrates. Traditional patterning methods using the AFM technique employ probe tips. Chemical bonds on the surface of the samples are broken by using the AFM tip creating lines with widths down to about 20 nm [8] corresponding to the tip radius. Self-assembly [9] is meanwhile a useful nanotechnology tool and is the conventional technique used in molecular biology. DNA is one of the biomaterials capable of self assembly allowing more capability and flexibility in fabrication resulting in efficient sensing elements [10]. Hector *et al.* [11] reported in 2007 that they utilized a new method of patterning called Shadow Nanolithography employing immobilized DNA strands. Recently, the use of DNA as a building block for nanosized materials has made it possible to extend this application to other branches of science such as nanoelectronics.

This paper describes a new very simple patterning method using DNA strands and involving patterning on thin film surfaces of Al deposited over Si absorbed layers using DNA strands. One of the important advantages of the DNA strands is their capability as a smart etching element for patterning with self assembly and reorientation in external electric fields. This method relies on chemical interactions between DNA and the Al surface to create nanometer scale patterns. As a result of this interaction, the traces of DNA strands would remain on the Al surface. According to AFM and FESEM techniques, this pattern corresponds to the dimensions of the DNA strands with lengths and diameters in the micron and nanometer scales, respectively. This type of patterning technique may be further optimized for utilization in microelectronics engineering, particularly for electrical and biosensors.

## 2. Experimental Section

**Materials:** DNA molecules from the plant *Boesenbergia rotunda* which belongs to the ginger family were extracted using the facilities available in-house at the Institute of Biological Science, University of Malaya. Sequence analysis indicated that the order of base pairs was as follows: A (22%), T (20%), G (35%), C (23%). The substrate used for etching was a p-type Si wafer (orientation <100>) of diameter (150.0 ± 0.1) mm, thickness (675 ± 25) μm and a resistivity of 1 to 10 Ω-cm bought from MEMC Electronic Materials. The Al wire used in thermal evaporation meanwhile was bought from the Kurt J. Lesker Company and had a diameter of 1 mm and 99.999% purity. High purity chemicals (NH_3_, H_2_O_2_, HF, HCl and acetone) were supplied by Sigma Aldrich and used directly. Deionized water was obtained using a Barnstead Company (Nanopure) water system.

**Figure 1.**
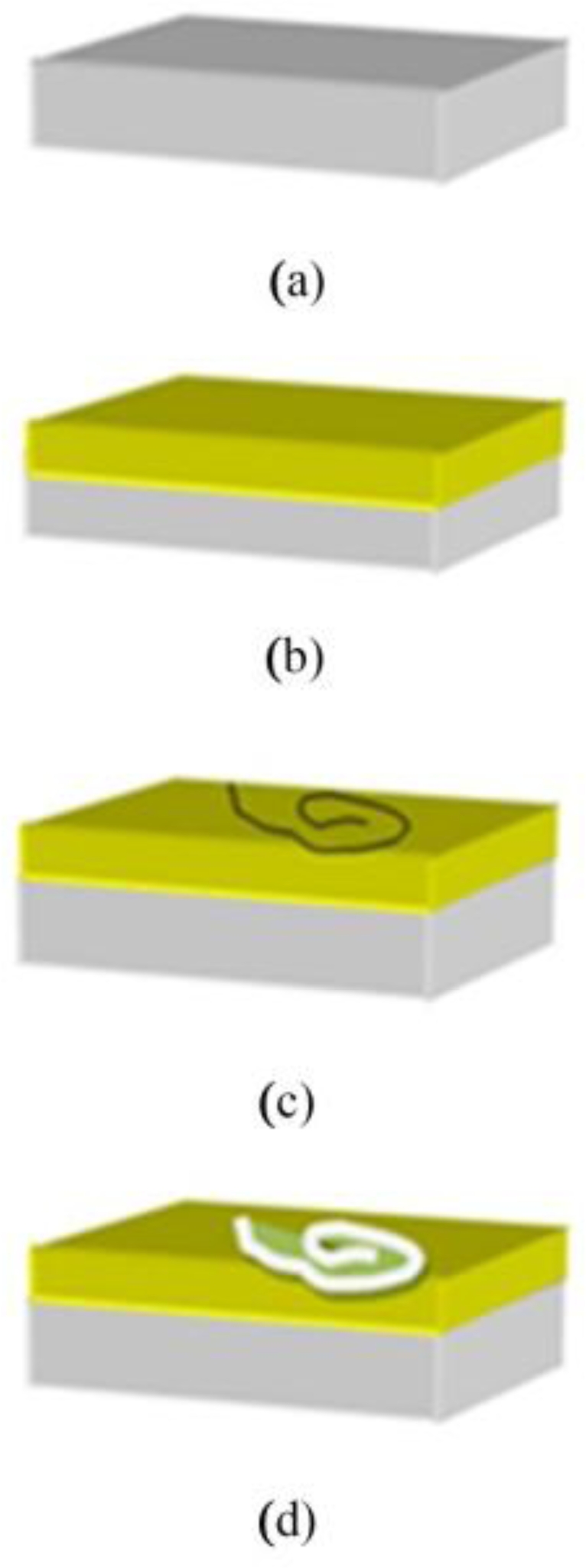
Schematic diagram showing the sample (**a**) cleaning silicon surface with standard method; (**b**) Aluminium deposition with thermal evaporation method; (**c**) DNA strands transfer on Al surface; (**d**) Removal of DNA strands off the surface reveals imprint of strands on surface.

**Experimental Setup:** The RCA (Reaction Chemical Agents) [12] process involving 10 min of boiling the p-type Si wafer in solutions of NH_3_, H_2_O_2_ and H_2_O (ratio 1:1:6) followed by boiling for 10 min in HCl, H_2_O_2_ and H_2_O (ratio 1:1:6) was performed to prepare the substrate. The native oxide on the frontal surface of the substrate was removed using HF and H_2_O solutions in the ratio of 1 to 10. Finally, it was rinsed in de-ionized water for 30 s (as shown as Figure 1(a)), followed by deposition of 100 nm of Al using thermal evaporation method (according to Figure 1(b)). The resulting thin film of Al thin film was then annealed for 30 min at 200 °C.

The DNA solution was first diluted to a suitable concentration of 0.01 (μg/μL) and allowed to flow along the Al surface using a micro syringe (Hamilton micro syringe) (as shown as Figure 1(c)). The prepared chip was then left exposed to the dry gas to let the liquid gradually and completely evaporate away. We control the duration of the interaction between DNA and Al thin film. This means that after 10, 20, 30 and 40 min of exposure of the DNA solution with the Al surface, the DNA strands are removed off the surface (as depicted as Figure 1(d)). The depths of the gaps formed by the DNA strands on Al are analyzed by the AFM and FESEM imaging techniques. The image shows a gap (less than 100 nm deep) formed by impact of DNA strands on the surface.

## 3. Results and Discussion

**Figure 2.**
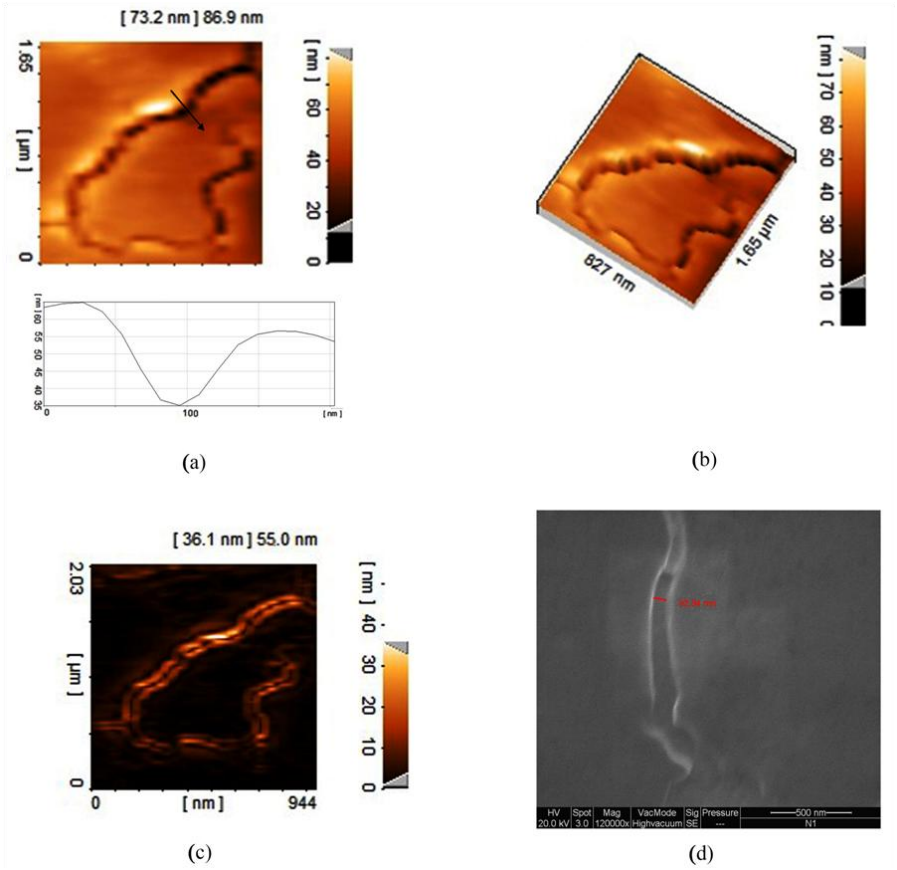
AFM imaging of bundled DNA strands effect n the Al thin film surface illustrate the formation of nano-gaps corresponding to the dimensions of the strands used in the experiment. (**a**) 2-Dimention image of surface; (**b**) depth profile in red arrow direction; (**c**) 3-Dimention image of (a); and (**d**) edge detection of (a).

**Figure 3.**
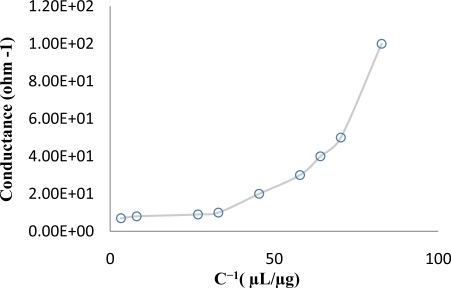
The conductivity *versus* reverse concentration of DNA strand (C^−1^) in aqueous environment in Au-DNA-Au structure at room temperature.

Chemical etching, achieved by the DNA strands absorbed on the Al surface, created nanometer scaled patterns as shown in the AFM images in Figure 2. Figure 2(a) shows a 2-D AFM image of the bundled DNA strands effect on the Al thin film surface that illustrates the formation of nano-gaps corresponding to the dimensions of the strands and the curve below Figure 2(a) illustrates depth profile in arrow direction for Figure 2(a) and 3-D image of Figure 2(a) is illustrated in Figure 2(b). Figure 2(c) shows edge detection of bundled DNA strands effect on the Al thin film surface and Figure 2(d) shows the FESEM imaging of distance between two edges of gap equated 82.94 nm. As a result of corrosion of Al thin film caused by the DNA strands, nanogaps were created due to the DNA scales.

The mechanism for the interaction between aluminium and DNA strands is not well understood but we have found that in an aqueous environment the interaction is strong and under dry conditions it is very poor. The interaction between molecules and water is due to the charge exchange then leads to changes in the charge distribution in the molecule and expulsion of charges from molecule to solvent. Since DNA as a big molecule also there is a charge exchange between the base pairs and water.

With purpose of investigating the moisture environment needed to create charge carriers in aqueous DNA we investigated the effect of humidity on carrier transportation in the Au-DNA-Au structure. The concentration of DNA C (unit of concentration (C) is μg/μL) is inversely proportional to the volume of water in solution (therefore the water volume have directly proportional to C^−1^) and according to Figure 3, an exponential increase in charge carrier in a moist environment is observed.

This phenomenon is because of the increased number of carriers for transport in the environment surrounding the DNA strands which increases the number of ions. As a result of these charge carriers, the π orbital symmetry will change and the electron density of DNA will redistribute [13]. Afterwards, the binding will change from covalent form to ionic form in aqueous situation.

In addition, aluminium is an electrochemically active material. There is very strong possibility that this interaction be caused by Al^3+^ ion binding with phosphate groups in the DNA strands. The ionization potential for a phosphate group as a smallest block of the DNA is small and ionization of H_2_PO_4_^−^ easily occurs so the free charge of DNA is from its sugar-phosphate backbone [14].

This carrier has the ability to interact with Al, at the interface of Al and DNA strands. The surface of the Al thin film in the interaction with this ionic buffer makes the etching process occur. Around a DNA strand, the concentration of charge is more than in other places. Thus the depth of etching on Al thin film is larger in the vicinity of DNA strands.

Another parameter that is important to create this effect is the time of exposure of the DNA strands to the Al thin film. Our observations indicate that during the first 20 min no detectable effect of the etching process on the Al thin film takes place. The interaction between the DNA strand and Al thin film, immediately after thermal evaporation is faster than when the Al is kept for a long time in the environment because of the growth of an oxide layer on the aluminium.

Also, the carrier in DNA strands, regarding their high density on the polar surface, likes to diffuse in the sublayers by a diffusion mechanism. Meanwhile, the aluminium atoms are very likely to move from one side with low concentration to other places with high concentration in a migration and diffusion manner.

**Figure 4.**
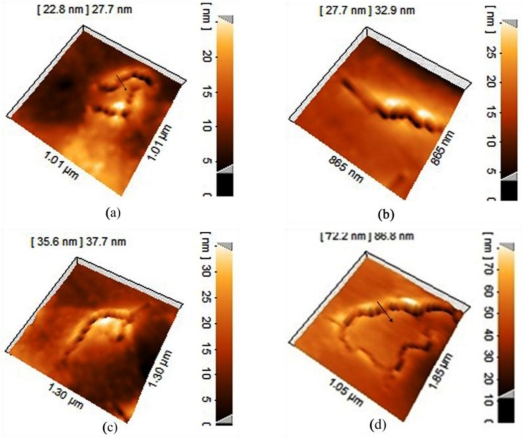
Depth profile of DNA strands remaining on the Al surface (**a**) after 10 min; (**b**) after 20 min; (**c**) after 30 min; and (**d**) after 40 min.

**Figure 5.**
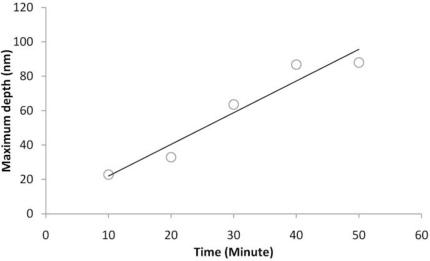
The maximum depth *versus* the reaction time.

As a result of this chemical interaction, an etching effect occurs; on the other hand, DNA traces remain on this substrate. Accordingly, by increasing the reaction time and conducting similar experiments for 10, 20, 30, 40 min durations, we achieved images with different depths. As shown in Figure 4, after a 10 min period, the depth created is around 22.8 nm, in 20 min it is around 27.7 nm and after 30 min it’s around 35.6 nm. Finally after a 10 min period lead to a gap with depth of around 72.2 nm. It is clearly deduced that the increase in the depth of a crack caused by DNA strands has a linear relationship with the time of interaction between DNA and the Al thin film (Figure 5). This will be saturated because of the Al thickness and DNA concentration.

**Figure 6.**
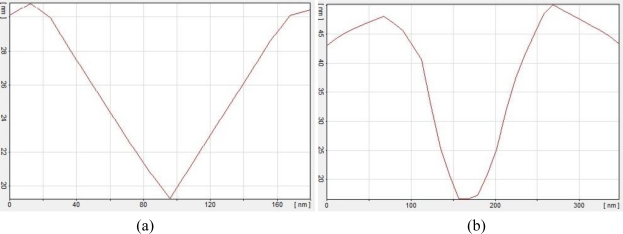
Side views of the formed gaps according Figure 4(a,d) along arrow. (**a**) is depth profile of Figure 4(a) in arrow direction and (**b**) is depth profile of Figure 4(d) in arrow direction.

Figure 6 presents side views of the formed gaps to provide some supplementary information for Figure 4. Figure 6(a,b) differ in two aspects: gap depth and shape of the valley. As seen in Figure 4(a) (indicated by the arrows) the valley bottom is sharp and pointed while the valley shown in Figure 4(d) is flat-bottomed. Figure 4(a) corresponds to a less deep gap than the one shown in Figure 4(d). Actually, the relevant gap depths of the valleys in Figure 6(a,b) are 30 and 50 nm. The maximum depth and configuration of the gaps can be related to the thickness of the deposited Al layer (it is less than 100 nm herein). Since DNA strands can only react with Al and are non-reactive toward silicon, the Al corrosion process along the vertical direction stops as soon as the DNA strands touch the silicon substrate surface. From this point on, corrosion along the horizontal axis enhances. In Figure 4(a), the Al-DNA interaction duration was not sufficient enough for DNA strands to reach the silicon wafer. Consequently, the gap tips are sharp. Yet, taking advantage of longer interaction duration, the DNA strands could cease their horizontal propagation.

## 4. Conclusions

A new method of fabricating nanometer scale patterns without the need for lithography was reported in this project. The DNA strands can be very important as nanostructure materials in nanotechnology and nanolithography systems. In this pioneering work, we have utilized these structures for nanopatterning using the principles of the chemical etching mechanism. Chemical interactions between the Al and DNA strands cause etching of the deposited Al thin film leaving imprints of the strands on the surface. The patterning achieved using the DNA strands as shown in this work to be a simple low-cost trajectory process. Further optimization based on the important method proposed may result in highly patterned substrates which could find variety of applications in microelectronics and nano-bioelectronics, especially in the fabrication of bio-sensors.
